# Using Clinical Cases to Restore Basic Science Immunology Knowledge in Physicians and Senior Medical Students

**DOI:** 10.3389/fimmu.2020.01756

**Published:** 2020-08-19

**Authors:** Mohammed Yousuf Karim

**Affiliations:** ^1^Acting Division Chief, Hematopathology, Sidra Medicine, Doha, Qatar; ^2^Assistant Professor in Clinical Pathology and Laboratory Medicine, Weill Cornell Medicine-Qatar, Ar-Rayyan, Qatar

**Keywords:** postgraduate, education, rheumatology, immunodeficiency, interactive, case-based learning

## Abstract

The majority of medical students and many physicians find basic science immunology confusing and the teaching of immunology to be uninteresting. Physicians undergoing training in a range of disciplines treat patients with immunological disease, including allergy/immunology and rheumatology. It is essential for senior medical students and physicians to understand the pathology of immune diseases and the pharmacology of immune interventions. In order to optimize this learning, underlying concepts of basic immunology need to be revised, or sometimes learned for the first time. Teachers may need to overcome baseline attitudinal negativity. Medical students and postgraduates are more able to relate to basic immunology if approached through a clinical route. Case presentations and case-based discussions are a familiar format for medical students and physicians, though typically utilized to enhance understanding of clinical presentation, investigation, and treatment. Hence, they may be more receptive to “difficult” immunology concepts when presented in a familiar teaching framework. Although there is data supporting case-based learning for basic immunology in medical students, there is little data in physicians. Extrapolating from the medical student literature, I devised a program of clinical cases for physicians whereby understanding the immunopathological basis of the condition and/or its immunological treatment was employed as a platform to appreciate the basic science immunology in more depth. A variety of cases were selected to illustrate different immunological topics. The sessions were small group and highly interactive in nature. As this programme has only recently been introduced, formal evaluation has yet to be concluded.

## Introduction

Immunology is considered difficult to understand, inducing trepidation in many medical students and physicians. It is essential to translate the subject in a comprehensible manner and disseminate knowledge in a practical fashion. The majority of medical students find basic immunology confusing (1, 2). Medical postgraduates in training are variously known in different countries as residents, fellows, junior doctors, and specialty registrars. In this article, I will use the terms *medical postgraduates* and *physicians* interchangeably. They are even more removed than medical students from their basic science immunology learning, and many also find immunology perplexing. The relevance of immunology to clinical practice cannot be underestimated. Physicians undergoing training in a wide range of disciplines treat patients with immunological disease, including allergy/immunology and rheumatology. Furthermore, there are curriculum requirements for basic science immunology for certain specialties (3, 4).

In clinical medicine, there is improvement in understanding, though by no means complete, regarding immunopathogenesis of many diseases. Consequently, an increasing number of immune therapies have been licensed, are used off-label, or are in clinical trials. Therefore, it is essential for senior medical students and physicians to understand the pathology of immune diseases and the pharmacology of immune interventions. In order to optimize this learning, underlying concepts of basic immunology need to be revised, or in some cases learned for the first time.

## Challenges

There is little data available in the literature regarding teaching of basic science immunology to medical postgraduates, in contrast to several research studies regarding medical students (1, 2, 5–10). In some cases, therefore, we may need to extrapolate to some degree from such studies of senior medical students, while recognizing the limitations of this approach, and considering important differences. We may also extrapolate from studies of other basic science topics, rather than specifically of immunology.

There are a number of challenges faced by both teachers and medical students and postgraduates in (re)learning basic science immunology. These are summarized in Table 1. There may be an underlying attitude toward immunology during medical school days; indeed, Dr. Amolak Bansal reported that 75% of medical students found immunology hard to understand, and only 1/3 found undergraduate immunology teaching to be interesting (1). Anecdotally, physician attitudes toward basic science immunology remain largely unchanged compared with their undergraduate days. The teacher may therefore have to already surmount potential baseline attitudinal negativity. Amongst clinical medical students, 29% identified pathology as the subject with the least practical application, compared with physiology (66%) (11). Students became more negative in their views regarding basic science courses with their seniority (12).

**Table 1:** Barriers to learning Immunology in senior medical students and postgraduates.

Pre-existing conceptions or misconceptions of Immunology as a “difficult” discipline
Variability of knowledge retained since undergraduate/early medical school teaching
Advances in knowledge since undergraduate/early medical school teaching
Tendency to “switch off” to basic science topics, as compared to “clinical” topics
Becoming overwhelmed by the complexity of pathways, and the number of new pathways
Ever increasing lists and lists of CD numbers, cell subsets, cytokines; curriculum-megaly
Inappropriate selection of Teachers and Lecturers
More removed than medical students from their basic science Immunology learning*

Row marked* applies only to medical postgraduates.

It is well-recognized that senior medical students forget a considerable amount of the basic science learned during the first two years of medical school (13–16). For example, in an older study of a traditional curriculum, retention of anatomy knowledge was comparable to that of nonsense syllables (14). However, perhaps surprisingly, physicians do not forget as much basic science as might be expected. In a long-term study, performance decreased from approximately 40%−45% correct answers for medical students to 30% correct answers for doctors after 24 years of practice (17). Although more removed than medical students from basic science immunology learning, medical postgraduates training in relevant specialities will be still be closer to medical school learning than consultant or attending physicians. Concepts of signal transduction, genetics, and molecular biology, which all overlap with immunology teaching, will not be so distant. For medical students, immunology is just one of many basic science subjects, and many senior students may consider it to be of limited relevance to their chosen future specialty. In contrast, medical postgraduates should prove more motivated and receptive, given the direct relevance of immunology to their chosen specialty.

Selection of appropriate teachers and lecturers is a critical challenge. Clinician lecturers may have insufficient up-to-date basic science knowledge, while basic science lecturers may find the clinical correlation difficult (2). Researchers may focus in too much depth on a specific pathway, or on their own research. In my own experience between teaching biomedical technologists, senior medical students, and physicians, the latter group struggles with the basic science aspects, while the technologists often find that the clinical jargon and abbreviations/acronyms are taken for granted. Overall, a balance of teachers is important, sometimes combining teachers of different academic/clinical backgrounds, which we have done for small group teaching.

## Implementation

### Rationale

Medical students and postgraduates are more able to relate to basic immunology if approached through a clinical route (1, 5, 6). Case presentations and case-based discussions are a familiar format for senior medical students and physicians, though typically they are utilized to enhance understanding of clinical presentation, investigation, and treatment. Hence, they may be more receptive to “difficult” immunology concepts when presented in a familiar teaching framework (6). Recall of basic science knowledge in clinical practice is enhanced by integration of basic science concepts with clinical content during medical school teaching (18–21). This approach has been used to good effect with senior medical students to better integrate basic science and clinical medicine (13, 18, 22, 23). In particular, Spencer et al. (13) recommended re-exposure to basic sciences in the final year of medical school to augment understanding of clinical medicine. Kulasegaram goes beyond the concept of curricular integration, with the notion of cognitive integration—the “integrated understanding of basic and clinical sciences within the mind of the individual learner” (24, 25).

Over 20 years ago in Australia, Dr. Amolak Bansal recommended the use of problem-specific learning and the emphasis on clinical relevance in immunology teaching for medical students (1). A Chinese study has shown the benefit of a small group patient-oriented problem-solving (POPS) system in comparison to traditional lectures in immunology (26). Eighty-eight of students preferred the POPS, which was reflected in significantly higher test scores in the POPS group compared with the lecture group. However, the authors concluded the limitation on a practical basis would be having sufficient teaching staff to implement the POPS system widely. While this could be a limiting factor for senior medical students, it would not be a constraint for postgraduates given the much smaller numbers of physicians training in immunological specialties. There is only limited data for case-based instruction in immunology for physicians. For example, there is evidence for physicians reverting to use of knowledge in basic biomedical science, i.e., working back from basics when encountering complex/difficult clinical cases (10). Simulation with a case of inborn error of immunity (IEI) has been used for 2nd year medical students, with a summative immunodeficiency objective structured clinical examination question to assess the students' recognition of an IEI and their clinical reasoning (27). Clinical correlation exercises have been used for medical students in an immunology/microbiology study to prioritize from a list of diagnostic tests, justify selection of these and any additional tests, and consider the differential diagnosis. Cases included HTLV-1-leukemia, myeloma, rheumatoid arthritis (RA), and systemic lupus erythematosus (SLE) (9). Stuart reported favorable impact in both student satisfaction and examination scores of oral case presentations compared with didactic lectures alone for undergraduate medical students (8). Sannathimmappa (7) reported positive influence in final year medical students for a case-based approach in immunology and microbiology.

### Implementation in Practice

Immunology teaching is relevant to a wide range of physicians, including those training in:

- Allergy/Immunology
- Clinical Microbiology
- Hematology-Oncology
- Immunopathology (Clinical Laboratory Immunology)
- Infectious Diseases
- Intensive Care Medicine (Critical Care Medicine)
- Nephrology (Renal Medicine)
- Neurology
- Pulmonology (Respiratory Medicine)
- Rheumatology
- Transplantation (organ-based, stem-cell)

Novack has recently described in detail the development of the case-based teaching of medical students (23). Extrapolating from the literature in medical students, I have introduced the case-based format into our immunology teaching programme for medical postgraduates. In order to overcome the physicians' pre-existing apprehension, I devised a programme of clinical cases where understanding the immunopathological basis of the condition and/or its immunological treatment could be used as a platform for understanding the basic science immunology in more depth. A variety of cases were selected to illustrate a range of different immunological topics. The balance of the cases can be altered depending on the medical specialty of the postgraduates. For example, a case repertoire with a focus more on autoimmunity would be more useful for rheumatology and nephrology, compared with a focus more on host defense for infectious diseases, clinical microbiology, and clinical immunology trainees. Cases can therefore include allergy, autoimmunity, immunodeficiency, and transplantation. By focusing on clinical cases matching the interest of the physician, we gain their attention; and then try to maintain it during the explanation and discussion of the underpinning basic science. The setting is small group teaching, and the cases are presented initially by the lecturer using PowerPoint® slides. The cases are interspersed with multiple choice and open questions for the physicians, deliberately rotating between the audience. The questions mainly focus on the scientific rather than clinical aspects of the cases. The questions provide the focus of the discussion and identify areas of pre-existing knowledge and learning needs. The setting is very interactive, and the session is planned and timed so that it relies on the contributions of the physicians. It is important to be as positive and encouraging as possible, and to avoid overwhelming the audience with a soup of CD numbers, and cytokines. During the course, the physicians are encouraged to bring their own cases—this very much augments their interest and enhances the learning opportunity. I have detailed some of the cases below.

Case 1: Allergy/Immunology/Rheumatology—Chronic mucocutaneous candidiasis

Although only a minority of immune disease has been demonstrated to have a monogenic basis, these genetic defects, in particular, can enable detailed explanation of the normal immune processes. A patient with chronic mucocutaneous candidiasis due to homozygous AIRE mutation, with multi-organ involvement, and multiple autoantibodies initially presented to the Pediatric Rheumatology service. This case was used to explore and contrast normal T-cell development and the acquisition of thymic (central) tolerance. The number of recognized IEIs is increasing at a dramatic rate. In 2017, the International Union of Immunological Societies noted 320 IEIs with single gene defects, whereas the 2019 version has 430 IEIs (28, 29). While this presents a challenge to clinicians to keep up with the literature, it also presents an excellent opportunity for case-based teaching of immunological mechanisms. Discussion of immunodysregulatory disorders such as IPEX (immune dysregulation, polyendocrinopathy, enteropathy, and X-linked) enhanced the explanation of peripheral tolerance and FoxP3+ T-regulatory cells. Up until a few years ago, the finding of autoimmunity and immunodeficiency in the same patient was considered paradoxical (30), and such cases represent an opportunity to illustrate the concept of immunodysregulation (31).

Case 2: Allergy/Immunology—Common variable immunodeficiency

Common variable immunodeficiency (CVID) is considered primarily an antibody deficiency disorder. However, there is a subgroup who also develop autoimmune manifestations such as cytopenia, inflammatory bowel disease, and interstitial lung disease. A number of underlying mutations have been demonstrated including in LRBA (lipopolysaccharide-responsive and beige-like anchor protein) and CTLA4 (cytotoxic T-lymphocyte antigen-4), a potent T-cell inhibitory receptor. LRBA colocalizes with CTLA4 in endosomal vesicles and LRBA deficiency increases CTLA4 turnover, resulting in reduced CTLA4 protein in FoxP3+ T-regulatory and activated conventional T-cells (32). The elucidation of the interaction of LRBA and CTLA4, and the mechanism for CTLA4 trafficking and control of immune responses, not only provided an explanation of the underlying pathogenesis, but lead to the off-label clinical use of CTLA4-immunoglobulin (CTLA4-Ig, abatacept) in CVID lung disease. This is an excellent example of where the basic immunology explains the clinical efficacy of the therapy.

Case 3: Rheumatology—CTLA4-Ig and rheumatoid arthritis

**Figure 1:**
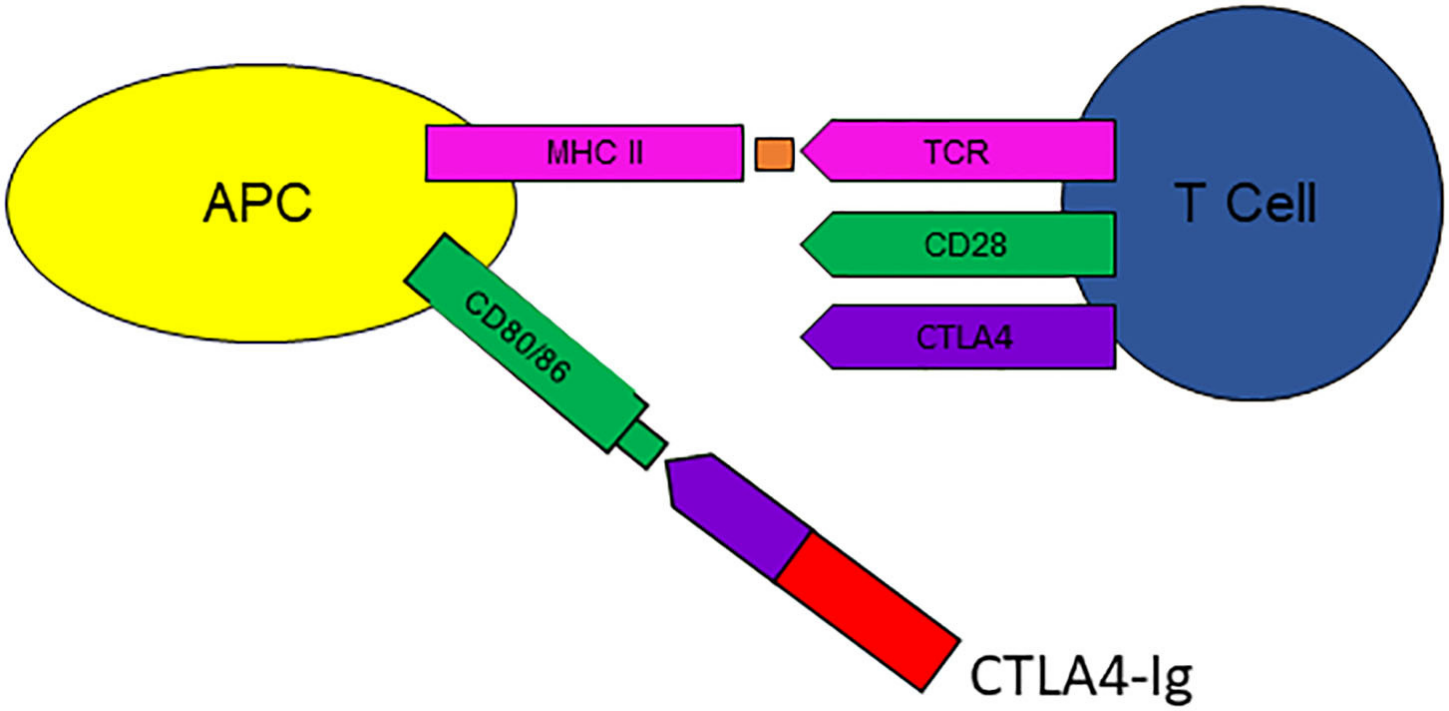
CTLA4-Ig (abatacept) binds to CD80/86 on antigen presenting cells (APC), interfering with activation of T-cells via CD28, thus preventing signal 2. Courtesy Zunairah Karim. TCR, T-cell receptor; MHC ii, major histocompatibility complex class II.

CTLA4-Ig is a fusion protein composed of the extracellular domain of CTLA4 with the Fc region of IgG1. It is primarily licensed for the treatment of RA, a common illness with a prevalence of 1%, and is currently in clinical trials in a number of other autoimmune diseases (AID). Presenting a case of its use in RA was an enabler for discussing the concepts of co-stimulation and signal 2, B-cell-T-cell, and antigen-presenting cell-T-cell interactions (Figure 1). The range of different co-stimulatory molecules was considered, and the importance of the CTLA-4:CD80/86 interaction to immune homeostasis—applying control of the T-cell immune response, and counterbalance to the activating interaction of CD28:CD80/86. Thus, exploring the mechanisms and use of immunological interventions can ameliorate basic science understanding.

Case 4: Nephrology/Neurology/Rheumatology—Systemic vasculitis treated with B-cell depletion therapy

B-cell depletion therapies (BCDT) are utilized in a range of AID, including multiple sclerosis, lupus nephritis, RA, and systemic vasculitis. The physicians were presented with a case of systemic vasculitis treated with multiple medications, including BCDT, over the course of the disease. The patient developed antibody deficiency, and the physicians were asked to work through the case. Thus, secondary hypogammaglobulinemia developing in patients treated with BCDT provided an opportunity to illustrate the normal process of B-cell development, and of antibody production. The questions posed to the audience included the molecular targets of BCDT, and establishing the mechanism of the antibody deficiency. The most commonly utilized BCDT, rituximab, targets CD20, which is restricted to B-cells, rather than plasma cells. Early reports in rituximab-treated patients showed that immunoglobulin levels were maintained, and hypogammaglobulinemia was considered unlikely because the long-lived plasma cells do not express CD20. However, more recent studies demonstrate that repeated BCDT cycles may lead to sustained B-memory cell depletion, with subsequent failure to replenish plasma cells.

In some cases, the knowledge gained in understanding immunopathogenesis was discovered more serendipitously than might be realized. This is important to emphasize, as there is a common misconception of the relative contribution that personalized medicine has made to date. In the 1990s, RA was largely considered a T-cell-mediated disease. Rituximab was introduced for B-cell lymphoma in 1997, and Prof Jo Edwards considered that this could also have efficacy in RA. His seminal article on BCDT in RA lead to the introduction of this treatment for a remarkable range of AID (33). However, the exact mechanism of BCDT in AID remains elusive: for example, in immune thrombocytopenia, the clinical improvement is much faster than any purported effects on autoantibodies.

Case 5: Infectious diseases, Clinical Microbiology—HIV infection

The central role of CD4 T-cells in protective immunity can be illustrated by HIV infection, which progresses to acquired immunodeficiency syndrome in some cases. The predisposition to opportunistic infections and malignancy occurs in relation to the CD4+ T-cell count, with the risk escalating as the CD4+ T-cell count reduces. This enabled a detailed discussion of the central role of CD4+ T-helper cells in adaptive immunity. The physicians could appreciate their critical role in the activation of CD8+ cytotoxic T-cells to counter viral infections, in the activation of macrophages to kill intracellular bacteria, and in providing help to B-cells to produce high affinity antibody.

Case 6: Nephrology/Rheumatology—SLE treated with anifrolumab

Physicians are welcome to bring their own cases, which they have worked up, or which they would like to understand more clearly with respect to the underlying basic immunology. This can be done in a flipped classroom model. A parallel can be drawn with medical physiology teaching for intensive care medicine residents, which they also considered a difficult subject to understand (34). Our sessions are in small groups and very interactive. Physicians are free to ask questions throughout, and a recent discussion point related to the use of “omics” data in the clinical management of the patient. This is likely to be an increasingly encountered scenario. The case was of severe SLE, which was introduced by one of the physicians in training. The limitations of measuring the interferon signature in patients with SLE were discussed following the recent anifrolumab (interferon-receptor antagonist) trial (35, 36). This allowed a detailed discussion of the multiple immune pathways which may be dysregulated in SLE, beyond the interferon axis, including both innate and adaptive immune responses.

## Discussion

Although the initial response of the medical postgraduates has been favorable, as this format has been recently introduced, it requires formal evaluation and detailed comparison, which remains to be undertaken. Currently, the physicians complete a simple 5-question evaluation of the programme, based on a Likert scale, with additional room for free comments. Although all the physicians sit the Fellowship examinations in their medical specialty, there is no formal examination specific to the programme. Preliminary evaluation in year 1 since the programme commenced has been undertaken. Eighty percent of physicians considered basic science immunology to be a difficult subject. Eighty percent felt that the case-based format was useful for understanding basic science immunology, with 60% considering this approach better than didactic lectures. We plan to formally evaluate and assess more detailed feedback from the programme over a longer period, and report this in a subsequent publication.

There are limitations to what this approach can achieve. There is a risk of oversimplification, in that the teaching focuses on the pathways and medications relevant to the clinical cases which are presented. There is a time limitation in terms of the number of cases presented, and the mechanisms which can be illustrated during the course. As a potential consequence, the physician may consider the immune system in terms of a set of disparate pathways, rather than appreciating the immune system as a whole, with its intricate coordination. To counter this, during the teaching programme, the links between parts of the immune response, particularly between innate and adaptive immunity, e.g., case 6, and between cell types, e.g., cases 3, 5 are emphasized. The importance of control of the immune response is illustrated, e.g., cases 1, 2.

Another risk is that medical students and postgraduates may concentrate on the clinical, rather than the immunological, aspects of the cases (22, 37, 38). However, the questions which act as the focal point of the discussion mainly focus on the scientific rather than clinical aspects of the cases. Senior medical students and physicians may focus their attention on pathways which appear more frequently in examination questions. Focusing on esoteric cases can give the erroneous impression that immunological conditions are rare and unimportant, with similar implication for the underlying basic science (39, 40). This is particularly important to avoid for senior medical students, who might otherwise be left with an enduring misconception regarding immune diseases. Hence, it is important to present a wide mixture of patients, in particular to include cases which the medical student or physician is likely to encounter during his/her routine clinical practice. The case mix can be varied according to the specialty of the physician in training. While learning basic science from IEIs has tremendous potential, it carries the caveat that many such IEIs are very rare, particularly in adult medicine. It is essential to emphasize the benefit to the physician/medical student in this context is not in learning the clinical details of the specific IEI, but in appreciating the underlying immunological mechanisms. Encouraging the clinicians to also bring cases to present themselves will challenge them to consider the underlying immunology, and personalize the learning experience for the clinician and their colleagues. There are online resources available which provide further examples of relevant cases, e.g., http://www.immunologyclinic.com/cases.asp (41). Development of further online resources is needed, and their utilization is encouraged.

While recognizing the above limitations, my impression is that the senior medical student and postgraduate will still gain valuable basic science knowledge which is relevant to his/her future medical, or surgical, practice. In conclusion, the understanding of basic science immunology is extremely important to senior medical students and a whole range of physicians in training. We consider that both senior medical students and postgraduates are more able to relate to basic immunology if approached through a clinical route. Although the initial response of the students has been positive, the efficacy of this case-based format requires formal evaluation and detailed comparison over a longer time period.

## Data Availability Statement

The original contributions presented in the study are included in the article/supplementary material, further inquiries can be directed to the corresponding author/s.

## Author Contributions

The author confirms being the sole contributor of this work and has approved it for publication.

## Conflict of Interest

The author declares that the research was conducted in the absence of any commercial or financial relationships that could be construed as a potential conflict of interest.
